# Plasma Levels of Oxidative Stress Markers, before and after *BoNT/A* Treatment, in Chronic Migraine

**DOI:** 10.3390/toxins11100608

**Published:** 2019-10-19

**Authors:** Elisa Dini, Sonia Mazzucchi, Ciro De Luca, Martina Cafalli, Lucia Chico, Annalisa Lo Gerfo, Gabriele Siciliano, Ubaldo Bonuccelli, Filippo Baldacci, Sara Gori

**Affiliations:** 1 Neurology Unit, Department of Clinical and Experimental Medicine, University of Pisa, 56126 Pisa, Italy; dini.elisa87@gmail.com (E.D.); mazzucchi.s@gmail.com (S.M.); delucaciro88@gmail.com (C.D.L.); l.chico@ao-pisa.toscana.it (L.C.); a.logerfo@ao-pisa.toscana.it (A.L.G.); gabriele.siciliano@unipi.it (G.S.); u.bonuccelli@med.unipi.it (U.B.); sara-gori@libero.it (S.G.); 2 Unit of Neurorehabilitation, Department of Medical Specialties, University Hospital of Pisa, 56126 Pisa, Italy; martinacafalli@gmail.com

**Keywords:** migraine, chronic migraine, medication overuse headache, BoNT/A, oxidative stress, biomarkers

## Abstract

The pathophysiological mechanisms of migraine transformation are debated. Modifications of plasma oxidative stress biomarkers have been described in chronic migraine. OnabotulintoxinA (BoNT/A) treatment, approved for chronic migraine prophylaxis, possibly reduces pain neurotransmitters release and oxidative stress products. Aims of our study were to investigate differences in the levels of selected plasmatic oxidative stress biomarkers (Advanced Oxidation Protein Products (AOPP), Ferric Reducing Antioxidant Power (FRAP), Thiolic Groups (SH)) comparing chronic migraineurs (CM) and healthy controls (HC). We also explored possible clinical and biochemical modifications in the CM group after six months of treatment with BoNT/A. At the baseline, we found higher values of AOPP (*p* < 0.001), and lower values of SH (*p* < 0.001) and FRAP (*p* = 0.005) in the CM group. At the six-month follow-up we found a reduction of AOPP (*p* < 0.001) and an increase of FRAP (*p* < 0.001) and SH (*p* = 0.023) within the CM group. BoNT/A treatment improved migraine symptoms in the CM group. We confirmed previous reports of imbalanced antioxidant mechanisms in chronic migraine showing lower antioxidant capacities in patients than controls. BoNT/A improved the levels of plasma oxidative stress biomarkers and confirmed its role as an effective prophylactic treatment for CM. Other studies should investigate the potential antioxidant properties of BoNT/A treatment.

## 1. Introduction

Migraine [1] is the most common neurological disorder, representing the third pathology for human prevalence and the seventh most disabling disease [2,3]. Its life-time course is variable. Some patients maintain a low frequency of attacks, while 3% transform into chronic migraineurs, with more than 15 days per month of headache, often with medication overuse [4,5]. OnabotulintoxinA (BoNT/A) was approved for chronic migraine treatment after two phase III studies demonstrated its efficacy as prophylaxis [6,7], even in the presence of painkiller abuse [8]. BoNT/A could hamper the release of substance P, calcitonin gene-related peptide (CGRP), and glutamate by the trigeminal/cervical nervous fibers, thus decreasing the liberation of pain neurotransmitters as well as oxidative stress products [9,10,11]. Further evidence supports the hypothesis of retrograde transport of BoNT/A along axons that may attenuate the central sensitization in the trigeminal ganglia and nucleus [12,13,14]. Although the pathophysiological mechanisms underlying migraine attacks and migraine transformation from an episodic to a chronic pattern are not fully understood, several data suggest that oxidative mitochondrial metabolism is altered in migraine patients. Modified levels of plasma oxidative stress biomarkers have been described in studies on either chronic migraine or neuropathic pain associated with central sensitization [15,16]. Literature data regarding oxidative stress biomarkers specific for migraine are heterogeneous. However, previous investigations reported that plasma levels of Advanced Oxidation Protein Products (AOPP), Ferric Reducing Antioxidant Power (FRAP), and Thiolic Groups (SH) showed significant differences between migraine patients and healthy controls (HC) [15].

Aims of our study are: (a) to investigate differences in the levels of the plasmatic oxidative stress biomarkers AOPP, FRAP, and SH comparing a population of chronic migraineurs (CM) with a group of HC; (b) to measure possible modifications of plasmatic concentrations of oxidative stress biomarkers in CM after a six-month period of treatment with BoNT/A; and (c) to measure possible modifications of migraine clinical features and outcomes after BoNT/A treatment, using appropriate and validated scales.

## 2. Results

Twenty-seven patients with a clinical diagnosis of chronic migraine with medication overuse and 27 HC matched for age and sex were recruited. Demographic data and results of biomarkers blood testing at baseline visit in the two groups are reported in Table 1. Significant differences between the two groups were found at T0 regarding plasmatic levels of AOPP (*p* < 0.001), SH (*p* < 0.001) and FRAP (*p* = 0.005). Clinical significant differences (*p* < 0.001) in the CM group were recorded at T1, with reduction of headache frequency, consumption of symptomatic drugs, FSS score, VNS score, HIT-6 score, ASC-12 score, GAD-7 score; no significant differences were obtained regarding the PHQ-9 score. Analysis of biomarker levels at T1 showed a significant increase of FRAP (*p* < 0.001) and SH (*p* = 0.023) and a significant reduction of AOPP (*p* < 0.001). Detailed results of clinical scales and biological assessments in the migraine group at T0 and T1 are reported in Table 2 and Table 3.

A comparison between plasmatic biomarkers levels measured in the CM group at T1 and values obtained in HC at T0 did not detect significant differences regarding AOPP and FRAP, whereas a significant difference was observed in SH levels (*p* = 0.01); detailed results are reported in Table 4.

## 3. Discussion

The results of the present study confirm previous observations of a close relationship between migraine and oxidative stress [15,17,18]. Impairment of antioxidant mechanisms in the group of migraine patients was measured employing the plasmatic oxidative stress biomarkers AOPP, FRAP, and SH. In a previous study on patients with a clinical diagnosis of chronic migraine with medication overuse, a significant reduction of FRAP and SH levels was reported compared to HC, whereas evaluation AOPP levels failed to demonstrate significant differences [15]. At the baseline evaluation we obtained significantly higher values of AOPP (*p* < 0.001) and lower values of SH (*p* < 0.001) and FRAP (*p* = 0.005) in the CM group, with respect to HC.

The complex mechanisms of action of BoNT/A on nociception include the block of CGRP release with consequent effects on peripheral sensitization in response to inflammation [19,20,21,22,23]. In order to evaluate possible modifications of plasmatic oxidative stress biomarkers after exposure to BoNT/A in the CM group we measured the levels of selected biomarkers after 6 months of treatment (T1). We detected a significant reduction of AOPP (*p* < 0.001) and increase of FRAP (*p* < 0.001) and SH (*p* = 0.023) with respect to baseline. Overall, according to our results, BoNT/A treatment seems to improve the functioning of antioxidant mechanisms in CM patients, with normalization of AOPP and FRAP levels after the 6-month observational period compared to HC. Median values of the SH biomarker, however, remained significantly lower of those measured in the HC group.

A further objective of the study was to investigate the effect of BoNT/A on clinical features, measured by means of specific scales and disease descriptors, including pain intensity (VNS), disability (HIT-6), presence of fatigue (FSS) or anxiety (GAD-7), drug consumption and headache days/month. As expected, BoNT/A treatment improved migraine symptoms in the CM group with a significant reduction (*p* < 0.001) of the scores of all parameters considered at the T1 evaluation, except for the PHQ–9 questionnaire [24].

The prospective evaluation of allodynia using the ASC-12 after the 6-month treatment with BoNT/A showed a significant reduction of the score (*p* < 0.001) from a median value of 5 at T0 (2.0-12.0), to a median value of 2 at T1 (0–6.0). Repeated episodes of migraine have a cumulative effect in inducing the development of central sensitization phenomena of the trigeminus-vascular system, which seems to exert its clinical expression in allodynia [25,26].

The results of the present study confirm previous reports of imbalanced antioxidant mechanisms in chronic migraine and medication overuse headache, with a reduction of antioxidant capacities in patients compared to controls [15,16,17,18]. BoNT/A treatment confirms its role as an effective prophylactic treatment for chronic migraine, not only reducing the number of days per month and drug consumption but also improving associated symptoms, particularly ictal allodynia. According to our data, BoNT/A treatment outcome could have indirect antioxidant properties, improving the levels of plasma oxidative stress biomarkers. More interesting, a BoNT/A direct antioxidant property could be speculated. Animal models of BoNT/A injection, in fact, demonstrated a reduction of intracellular accumulation of reactive oxygen species (ROS) and the activation of chemoprotective and antioxidant genes such as glutathione S-transferase [27,28]. Comparative studies with other molecules approved for chronic migraine and medication overuse headache are mandatory to corroborate this hypothesis.

However, our study needs some caveats. First, the sample size is relatively small. As a consequence, sub-population analyses, such as BoNT/A responders versus non-responders or triptan overuse versus nonsteroidal anti-inflammatory drugs (NSAIDs) overuse are not possible. The group of episodic migraineurs could not be included in the treatment, as BoNT/A is not approved for episodic migraine [6,7]. The respective weight of chronic migraine and medication overuse to oxidative stress impairment could not be assessed, because all patients have both diagnoses. The role of triptans or NSAIDs consumption on the described reduced antioxidant activity in those patients [15], as well as chronic migraine per se, could not be investigated in this clinical setting. Moreover, we did not search for potential sex differences. Finally, we did not include a group of migraineurs with aura and the follow-up is relatively short compared to the natural history of the disease. Additionally, plasma biomarkers level of episodic migraineurs were not collected, thus, a comparison between antioxidant levels in episodic and chronic patients could not be performed.

Further studies will be necessary to understand the pathophysiological mechanisms leading to chronic migraine and to determine the effective relationship between migraine and antioxidant mechanisms modifications, clarifying the possible role of BoNT/A in this field.

## 4. Materials and Methods

### 4.1. Study Population

A prospective study was carried out. We recruited consecutive outpatients of the Headache Centre of the University of Pisa, from October 2017 to March 2019, with the following enlisted inclusion criteria (IC) and exclusion criteria (EC). IC: (1) age ≥ 18 years; (2) normal neurologic evaluation; (3) brain MRI examination without pathological findings; and (4) absence of migraine preventive treatment for at least 3 months before study inclusion; (5) patient fulfilling both chronic migraine and medication-overuse headache according to ICHD-3 diagnostic criteria [1]; EC: (1) comorbid medical disorders and treatments for chronic systemic diseases; (2) patients with headache attacks fulfilling the criteria for Migraine with aura, according to ICHD-3. The second EC was determined to obtain a more homogeneous sample and only patients affected by migraine without aura were included, thus excluding subjects presenting migraine with aura ab initio. The overused medications were also limited to NSAIDs and triptans. As far as controls are concerned, healthy subjects were recruited among patients’ family members, not genetically related, and hospital workers. Individuals with headache history, as assessed through a clinical interview, were excluded; additionally, subjects with a positive history of medical disorders or with chronic medication use were excluded from the present study. All patients were BoNT/A-naïve and were not enrolled in previous clinical studies on migraine. This study has been performed in accordance with the Declaration of Helsinki and it was approved by the local ethics committee (Comitato Etico Area Vasta Nord Ovest – CEAVNO, Sezione Autonoma del Comitato Etico Regionale per la Sperimentazione Clinica, Via Roma 67, 56126, Pisa, Italy). All patients provided valid written, informed consent before study inclusion. The approval code and date: ID14518 in 19 February 2019.

### 4.2. Clinical Evaluation

Patients underwent three visits at intervals of three months: baseline (T0), 3-month, and 6-month (T1) evaluation. At the end of each visit (T0, 3-month, and T1) BoNT/A was administered according to PREEMPT protocol [6,7].

Clinical features of migraine were measured by means of appropriate scales [29,30] during T0 and T1 visits: Verbal Numeric Scale (VNS) to calculate pain intensity, Headache Impact Test (HIT-6) for the assessment of related disability, Fatigue Severity Scale (FSS) that measures fatigue-associated disability, Allodynia Symptoms Check-list 12 (ASC-12) for a specific measure of ictal allodynia, Generalized Anxiety disorder (GAD – 7) and Patient Health Questionnaire (PHQ – 9), indicators of anxiety and mood disorders. Clinical assessment during 3-month visit was conducted with an interview without the abovementioned questionnaires.

The patients were asked to record headache frequency on their diary before T0 and T1 visits, to measure the number of headache days per month.

### 4.3. Biochemical Evaluation

Blood samples were collected during T0 and T1 visit for CM group and only at T0 for the HC group. Blood venous samples were collected in the early morning, shortly after a light breakfast. Samples were centrifuged at 2500× *g* for 10 min at 4 °C within 2 h after collection and analyzed within 1 month after collection. Plasmatic levels of AOPP, FRAP, and SH were determined in patients and HC as described elsewhere [15]. All procedures were performed at our Neurology Unit Laboratory.

AOPP are markers of oxidative damage to proteins and include reaction products of the plasma proteins produced by the myeloperoxidase enzyme. Results were expressed as nmol/mL of chloramine equivalents.

FRAP is a biomarker that estimates the antioxidant power and measures non-enzymatic antioxidant properties. The data were expressed as mmol/L.

A further component of plasma antioxidant barrier is represented by total thiol groups. The sulfhydryl groups (SH) of plasma molecules can oppose the propagation of oxidative processes. Data were expressed as mol/L.

### 4.4. Statistical Analysis

The quantitative variables were not normally distributed. The continuous variables are expressed as Average, Standard Deviation, Median and Interquartile Interval (IQ). The categorical variables are expressed as frequency percentages. The comparison between non-parametric quantitative variables was performed employing the Wilcoxon test to compare the difference at T0 and T1 of the quantitative variables within the migraine population. Mann -Whitney U test was used to compare the difference of the quantitative variables between the migraineurs and controls. The comparison between categorical dichotomous variables was performed by the chi-square test with continuity correction. SPSS version 24.0 for Windows was used for statistical analyses.

## Figures and Tables

**Table 1:** Demographic descriptors and biomarkers values of oxidative stress between HC and CM groups at T0. Quantitative variables are expressed in terms of median and interquartile, the categorical variables are expressed as a percentage.

Variable	HC Group	CM Group	*p*-Value
Sex F/M (percentage)	F = 21 (77.8%) M = 6 (22.2%)	F = 21 (77.8%) M = 6 (22.2%)	NS
Age (years) (Median, IQ)	54 (45–59)	53 (44–58)	NS
AOPP nmol/L (Median, IQ)	119.1 (101.3–189.2)	258.0 * (192.5–373.1)	<0.001
FRAP mmol/L (Median, IQ)	0.817 * (0.646–0.861)	0.612 (0.559–0.557)	=0.005
SH μmol/L (Median, IQ)	0.456 * (0.395–0.549)	0.297 (0.237–0.381)	<0.001

AOPP: advanced oxidation protein products; CM: chronic migraine; FRAP: ferric-reducing antioxidant power; HC: Healthy Controls; SH: Thiolic Groups; IQ: interquartile range; NS: not statistically significant. *: significantly higher values (*p* < 0.05).

**Table 2:** Comparison between plasmatic values of oxidative stress biomarkers (AOPP, FRAP, and SH) at T0 (baseline) and T1 (6-month follow-up) in the CM group.

Variable	T0	T1	*p*-Value
AOPP nmol/L (Median, IQ)	258.0 (192.5–373.1) *	202.8 (87.3–253.3)	<0.001
FRAP mmol/L (Median, IQ)	0.612 (0.559–0.557)	0.752 (0.568–0.918) *	<0.001
SH μmol/L (Median, IQ)	0.297 (0.237–0.381)	0.368 (0.272–0.460) *	=0.023

AOPP: advanced oxidation protein products; FRAP: ferric-reducing antioxidant power; IQ: interquartile range; HC: Healthy Controls; CM: chronic migraine; SH: Thiolic Groups. *: significantly higher values (*p* < 0.05).

**Table 3:** Comparison between T0 and T1 regarding clinical features and scores obtained for each migraine scale in the CM group.

Variable	T0 Median (IQ)	T1 Median (IQ)	*p*-Value
Headache frequency (days/month)	25.0 * (20.0–30.0)	15.5 (11.5–25.0)	< 0.001
Symptomatics/per month	25.0 * (20.0–30.0)	15.0 (10.0–25.0)	< 0.001
FSS	47.0 * (38.0–53.0)	28.0 (27.0–32.0)	< 0.001
VNS	8.0 * (8.0–10.0)	5.0 (5.0–7.0)	< 0.001
HIT-6	66.0 * (64.0–73.0)	56.0 (51.0–63.0)	< 0.001
ASC-12	5.0 * (2.0–12.0)	2.0 (0–6.0)	< 0.001
GAD 7	10.0 * (7.0–16.0)	9.0 (6.0–13.0)	< 0.001
PHQ 9	9.0 (6.0–15.0)	9.0 (6.0–14.0)	NS

ASC-12: Allodynia Symptoms Checklist 12, FSS: Fatigue Severity Scale; GAD–7: Generalized Anxiety Disorder; HIT-6: Headache Impact Test; IQ: interquartile range; PHQ–9: Patient Health Questionnaire; VNS: Verbal Numeric Scale; NS: not statistically significant. *: significantly higher values (*p* < 0.05).

**Table 4:** Comparison between plasmatic values of oxidative stress biomarkers (AOPP, FRAP and SH) in HC Group and CM group at T1.

Variable	HC Group	CM Group T1	*p*-Value
AOPP nmol/L (Median, IQ)	119.1 (101.3–189.2)	202.8 (87.3–253.3)	NS
FRAP mmol/L (Median, IQ)	0.817 (0.646–0.861)	0.752 (0.568–0.918)	NS
SH μmol/L (Median, IQ)	0.456 * (0.395–0.549)	0.368 (0.272–0.460)	= 0.01

AOPP: advanced oxidation protein products; FRAP: ferric-reducing antioxidant power; IQ: interquartile range; HC: Healthy Controls; CM: chronic migraine; SH: Thiolic Groups. *: significantly higher values (*p* < 0.05).
